# Cross-cultural adaptation and validation of the Arabic version of the BACS scale (the brief assessment of cognition in schizophrenia) among chronic schizophrenic inpatients

**DOI:** 10.1186/s12888-021-03228-9

**Published:** 2021-05-01

**Authors:** Chadia Haddad, Pascale Salameh, Souheil Hallit, Sahar Obeid, Georges Haddad, Jean-Pierre Clément, Benjamin Calvet

**Affiliations:** 1 INSERM, University Limoges, CH Esquirol, IRD, U1094 Tropical Neuroepidemiology, Neurology, Limoges, France; 2 Pôle Universitaire de Psychiatrie de l’Adulte, de l’Agée et d’Addictologie, Centre Hospitalier Esquirol, 87000 Limoges, France; 3 Research Department, Psychiatric Hospital of the Cross, Jal Eddib, Lebanon; 4 INSPECT-LB: Institut National de Sante Publique, Epidémiologie Clinique et Toxicologie, Beirut, Lebanon; 5 Faculty of Pharmacy, Lebanese University, Beirut, Lebanon; 6 Medical School, University of Nicosia, Nicosia, Cyprus; 7 Faculty of Medicine and Medical Sciences, Holy Spirit University of Kaslik (USEK), Jounieh, Lebanon; 8 Faculty of Arts and Sciences, Holy Spirit University of Kaslik (USEK), Jounieh, Lebanon; 9 Centre mémoire De Ressources et De Recherche du Limousin, Centre Hospitalier Esquirol, 87000 Limoges, France

**Keywords:** Schizophrenia, Arabic, Cognition, Assessment, Validation, BACS

## Abstract

**Background:**

Assessment of cognitive disorders in schizophrenia is becoming a part of clinical and research practice by using batteries that differ widely in their content. The Brief Assessment of Cognition in Schizophrenia (BACS) was developed to cover the main cognitive deficits of schizophrenia. The objective of this study was to assess concurrent validity of the Arabic version of the BACS with a standard neurocognitive battery of tests in Lebanese patients with schizophrenia and healthy controls.

**Methods:**

A sample of 120 stable inpatients diagnosed with schizophrenia and 60 healthy controls received the Arabic version of the BACS in a first session, and a standard battery in a second session.

**Results:**

Mean duration of completion for the BACS was 31.2 ± 5.4 min in patients with schizophrenia. All tests demonstrated significant differences between controls and patients (*p* < .01). Principal components analysis demonstrated that a one-factor solution best fits our dataset (64.8% of the variance). High Cronbach alpha was found (.85). The BACS composite scores were significantly correlated with the standard battery composite scores in patients (*r* = .73, *p* < .001) and healthy controls (*r* = .78, *p* < .001). Also, correlation analysis between the BACS sub-scores and the standard battery sub-scores showed significant results (*p* < .05).

**Conclusion:**

Results showed that the Arabic version of the BACS demonstrated high ability to discriminate patients with schizophrenia from healthy controls and it is a useful tool for assessing cognition in patients with schizophrenia and could be used in clinical practice in Lebanon.

## Background

Patients with schizophrenia have deficiencies in a variety of cognitive functions, including verbal memory, working memory, motor speed, attention, executive functions, and verbal fluency, which affect up to 75% of patients [1]. Social cognition is also impaired in people with schizophrenia. They also have difficulties to perceive social input, which can lead to misunderstandings of others’ social intentions, social withdrawal, and impaired daily social functioning [2]. Cognitive function of patients with schizophrenia is lower by 1.5 to 2 SD (standard deviation) on several dimensions compared with results of healthy controls [3]. These cognitive impairments appear to be linked to social and functional outcomes and seemed to be independent of positive and negative symptoms as well as psychotic treatment [4, 5]. Neurocognition was strongly correlated with daily functioning in a study of 921 patients with schizophrenia living in the Italian culture [6]. Another study of 921 people with schizophrenia showed that social cognition, neurocognition, resilience, and real-life functionality was both stable and independent structures [7]. Higher neurocognitive abilities were correlated with improved daily functioning in a recent multicenter prospective study involving 618 patients with schizophrenia from 24 Italian university psychiatric clinics or mental health departments [8]. Therefore, in patients with schizophrenia, cognitive testing is also one of the best markers of their functional and social prognosis.

Numerous neurocognitive batteries have been established to determine cognitive dysfunction in schizophrenia patients [9]. For example, the “MATRICS (Measurement and Treatment Research to Improve Cognition in Schizophrenia)” [10], the “RBANS (Repeatable Battery for the Assessment of Neuropsychological Status)” [11] and the “CANTAB (Cambridge Neuropsychological Test Automated Battery) batteries” [12]. However, the majority of them are long and complicated, assessing the patient’s entire neuropsychological profile and taking several hours to complete [13]. The availability of a brief and easy tool for evaluating cognitive function in schizophrenia patients could help clinicians making recommendations on future therapy and antipsychotic drug adaptation, as well as researchers evaluating cognitive changes during clinical trials.

The “Brief Assessment of Cognition in Schizophrenia (BACS)” is a reliable and efficient test battery that evaluates the major cognitive domains impaired in schizophrenia, including verbal memory, working memory, speed of information processing, motor speed, verbal fluency, and executive functions [14]. The BACS was created to be easily used by medical professionals such as psychiatric nurses, clinicians, psychologists, social workers, psychiatrists, and other mental health professionals [14]. The test session lasts over 35 min, with just a few minutes left over for scoring compared to more than 2 h for a standard cognitive battery [14].

The original version of the BACS was validated on a group of 150 schizophrenia patients and a sample of 50 stable controls and showed a good psychometric properties. A high test–retest reliability was found with an intraclass correlation coefficient ≥ 0.79 and the BACS total score was strongly correlated with the standard battery score for both patients (*r* = 0.79) and controls (*r* = 0.90) [14]. The BACS has been translated and validated in more than 30 languages, including English [14], French [15], German [16], Spanish [17], Brazilian [18], Chinese [19], Japanese [20], Persian [21] and Italian [22]. Compared to a standard cognitive battery, these versions showed adequate reliability and concurrent validity.

Few studies have been done in the Arab countries that evaluate the cognitive impairment in patients with schizophrenia [23–25]. In Lebanon, some studies evaluated cognitive function in older people [26–28]. However, no study was done to assess cognitive functions in individuals with neurological or psychiatric diseases. Clinicians had limited options, since they had to rely on untranslated assessments or translations that had not been validated. Some neuropsychological batteries had been used locally among patients with schizophrenia to assess their cognition such as the Wechsler Adult Intelligence Scale (WAIS) and the RBANS. Therefore, the adaptation and validation of the BACS into Arabic would help researchers in assessing cognitive impairment in schizophrenia and guide clinical decisions on cognitive interventions and rehabilitation. Thus, the objective of this study was to evaluate the concurrent validity of the Arabic version of the BACS with a standard neurocognitive battery of tests in Lebanese schizophrenia patients and healthy controls.

## Methods

### Study design and participants

A cross-sectional study was performed at the “Psychiatric Hospital of the Cross – Lebanon (HPC)”, between July 2019 and March 2020. The study enrolled 120 inpatients diagnosed with schizophrenia and schizoaffective disorders and 60 healthy controls, matched for age, education and sex. The inclusion criteria for patients were as follows: inpatients aged between 18 and 60 years; having an educational level over 5 years, meeting the “DSM-5 criteria (Diagnostic and Statistical Manual of Mental Disorders, fifth edition)” for schizophrenia and schizoaffective disorders; being treated with antipsychotics medication and clinically stable. The diagnosis was made by a clinical interview with the treating psychiatrist using the DSM-5 criteria. The clinical stability of the patients was defined as: “the period during which psychotic symptoms are less severe and the patient is on adequate treatment for at least last 6 months and did not require any increase in dose of antipsychotic medication over last 3 months” [29]. Inclusion in the patient group was not based on any particular prescription requirements. The healthy individuals were recruited from the staff of the HPC hospital who met the following criterion: absence of any mental and psychiatric disorders. Brain damage and neurological disorders or current substance use disorder were among the exclusion factors for all participants that would influence cognitive performance.

**Fig. 1:**
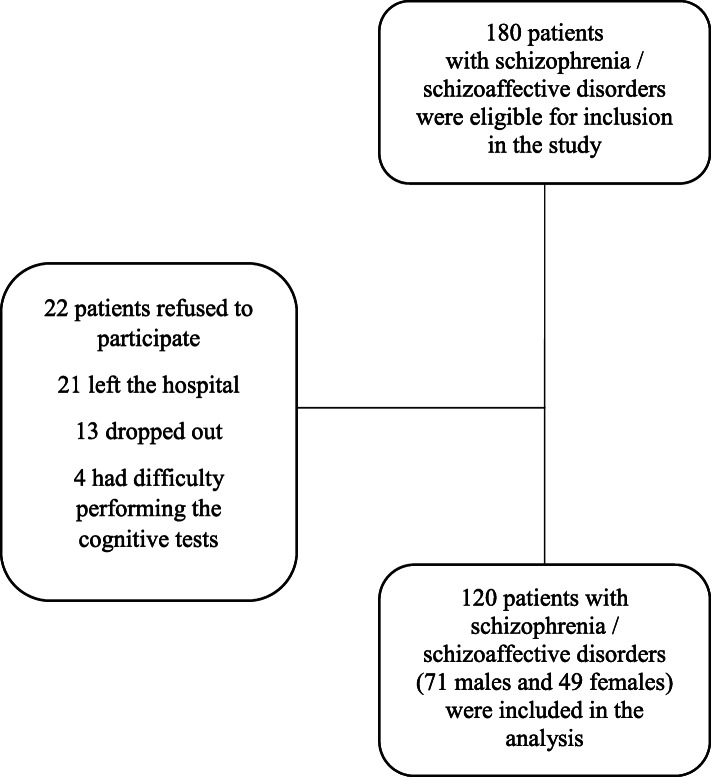
Enrollment of inpatients with schizophrenia

Based on a list generated from the hospital’s computer software, out of 180 patients selected according to the inclusion criteria, 120 patients (71 males and 49 females) were included. Sixty patients were excluded (40 males and 20 females) for the following reasons: 22 patients refused to participate, 21 left the hospital, 13 refused to continue the assessment and 4 had difficulty performing the cognitive tests (Fig. 1). Participants were asked to sign a written informed consent form without receiving any monetary reward if they wanted to participate in the study.

### Sample size calculation

In order to calculate the minimum sample size needed for our research, the Gpower 3.1.9.2 software was used with a power of 80% (1-β = 0.8) and an error α of 0.05, an effect size of 0.47 was calculated based on the original study done by Keefe et al. [14]. The BACS battery composite score was shown to be strongly related to a standard battery composite scores of both schizophrenia patients (*r* = 0.76) and stable controls (*r* = 0.90) in the study of Keefe et al. [14]. Taking into consideration a ratio of 2:1 in each group, the results showed that the minimal sample size needed was 164 (55 in the healthy control group and 109 in the patient group).

### Adaptation and assessment procedure

The Arabic version of the BACS has already been translated from English into classical Arabic language by Ateeq and collaborators using the standard translation/back-translation method among 33 patients with schizophrenia from Riyadh city (unpublished study). Pr. Richard Keefe sent us the Arabic version, which was reviewed and accepted by the University of Duke Medical Center’s Department of Psychiatry and Behavioral Sciences.

Demographic variables (age, sex, marital status, educational level and monthly income) and clinical information of the participants (diagnosis, duration of illness in years, duration of hospitalization in years, number of hospitalizations, medication intakes, and family history of mental disorders) were collected from medical files. The socioeconomic status was divided into four levels (no income, low (< 1.000 USD), intermediate (1.000–2.000 USD), high (> 2.000USD)) and the education level was divided into three levels (complementary level (> 5 years), secondary level (> 9 years) and university level (> 12 years)). Also, the dose of medications used was assessed by the chlorpromazine equivalent dose [30].

Two independent psychologists (one specialized in the administration of the BACS and the other trained in the use of the standard battery) assessed participants on two different days in less than 2 weeks between the two evaluations (mean difference between the 2 days: 5.67 days). The two evaluators were blind to each other, there is no memorization bias in order to avoid confounding factors. There were two versions of the BACS (version A and version B). These versions have the same tests with alternate forms. In our study we used version A. The BACS version A was used for the first test session, and the standard battery was used for the second session.

The BACS, included the following tests in the administered order:

*“Verbal memory – list learning”*

Participants were given 15 words and asked to remember as many as they could. This process was carried out five times. The number of words remembered per trial, in any order, was used to assess performance (with a range between 0 and 75).

*“Working memory – digit sequencing task”*

Participants were presented with increasing-length clusters of numbers. They were instructed to order the numbers from lowest to highest for the experimenter. The number of correct responses was used to assess efficiency (with a range between 0 and 28).

*“Motor speed – token motor task”*

Participants were given 100 plastic tokens and instructed to insert two at a time into a jar as quickly as possible. The limited time required to complete the task was 60-s. Performance was calculated in terms of the amount of tokens correctly inserted into the jar (with a range between 0 and 100 at the final outcome).

*“Verbal fluency”*

“Category instances”: Participants had 60 s to list as many words as they could from a certain category (animals).

“Controlled oral words association test”: participants were given 60 s in two separate trials to produce as many words as possible that started with a certain letter, T, R. The letters “م” (similar to M in English) and letter “ج” (similar to G in English) were used since these letters in Arabic had as much word redundancy as the letters T and R in English. The overall test score refers to the number of words generated correctly within 60 s. The total score for the verbal fluency test refers to the sum of the three trials. Higher scores reflect a better performance.

*“Attention and speed information processing – symbol coding”*

During 90 s, participants were asked to write the numerals 1–9 as matches to symbols on an answer sheet as quickly as possible. The number of correct numerals was used to assess results (with a range between 0 and 110).

*“Executive functions – Tower of London”.*

Participants were shown two images at the same time. Three balls of various colors were placed on three pegs in each picture, with the balls in a unique configuration in each picture. Subjects were asked how many times the balls in one picture had to be rotated in order for the configuration of balls in the other, opposite picture to be equal. A total of 20 trials were conducted. The items were of varying complexity, with a general trend toward more challenging items as the game progressed. If patients completed correctly all 20 trials, they were given two more trials that were more complicated. The number of correct answers was used to assess efficiency (with a range between 0 and 22).

*Standard battery*

The standard battery consisted of tests designed to examine the same structures as the BACS. The tests and their respective constructs are described in the following order: “16 item Free and Cued Recall test (RL/RI-16) (verbal memory)”, “Forward and Backward Digit Span Sequencing from the WAIS-IV (working memory)” [31], “Trail Making Test A (TMT-A) (motor speed)”, “Controlled Oral Word Association Test” (letter “ب” (similar to B in English) and “ف” (similar to F in English), “Category Instances (Fruit category) (verbal fluency)”, “Digit Symbol Coding from the WAIS-IV (attention and speed of information processing)” [31] and “Block Design Test from the WAIS-IV (reasoning and problem solving)” [31].

### Data analysis

The SPSS software version 25 was used to conduct the data analysis. The Shapiro Wilk test was used to verify the normality distribution of the BACS scale. The major dependent variable was normally distributed. A descriptive analysis was carried out where quantitative variables were expressed as means and standard deviations, while categorical variables were expressed as absolute frequencies and percentages. In order to evaluate categorical variables, the Chi-square and Fisher exact tests were used while to compare continuous variables between groups the Student T-test was used.

The Arabic-BACS composite scores and the standard neurocognitive battery were determined by averaging all the subscales of each instrument and converting them to z-scores. Pearson correlations with the equivalent scales of the standard battery were used to assess the concurrent validity of the Arabic-BACS subscales. The principal component analysis was used to assess the construct validity of the BACS instrument. The Kaiser-Meyer-Olkin measure of sampling adequacy and Bartlett’s test of sphericity were determined to ensure the model’s adequacy. The number of components to extract was determined using the scree plot procedure and factors with eigenvalues values greater than one were kept [32]. Only items with a factor loading greater than 0.4 were taken into account [33]. Moreover, the Arabic-BACS’ internal consistency was tested using Cronbach’s alpha. Face validity was investigated using a Student T-test to compare subtest scores between patients and controls. Threshold for discrimination between schizophrenic cases and controls was determined, in addition to sensitivity and specificity, using “receiver–operator characteristics (ROC)” curves, where all schizophrenic patients were considered “cases” and all controls “non-cases”. Statistical significance was described as a *P*-value of less than 0.05.

## Results

### Sociodemographic characteristics

Sociodemographic characteristics of the participants are described in Table 1. Mean age of patients with schizophrenia was 48.4 ± 7.6 years, with 59.2% males. The majority (81.9%) were single, with low monthly income (52.6%), 50% have a secondary level of education and 36.5% have a family history of psychiatric illness. Mean duration of illness and hospitalization were 20.6 ± 12.4 and 12.4 ± 8.5 years respectively. The healthy control group was matched with the schizophrenia group according to sex, education level and age. The two groups differ on marital status, monthly income and family history of psychiatric illness. Married participants with high monthly income and without any psychiatric illness were found in the control group as compared to the patient group.

**Table 1:** Sociodemographic characteristics of the sample

	Patients with Schizophrenia (***N*** = 120)	Healthy controls (***N*** = 60)	***p***-value
	Frequency (%)	Frequency (%)	
**Sex**			
Male	71 (59.2%)	36 (60.0%)	0.91
Female	49 (40.8%)	24 (40.0%)	
**Education level**			
Complementary	41 (34.2%)	21 (35.0%)	0.73
Secondary	60 (50.0%)	27 (45.0%)	
University	19 (15.8%)	12 (20.0%)	
**Marital Status**			
Single	95 (81.9%)	6 (10.0%)	< 0.001
Married	9 (7.8%)	52 (86.7%)	
Widowed	2 (1.7%)	0 (0.0%)	
Divorced	10 (8.6%)	2 (3.3%)	
**Monthly income**			
No income	27 (23.3%)	0 (0.0%)	< 0.001
< 1000 $	61 (52.6%)	39 (67.2%)	
1000–2000 $	26 (22.4%)	12 (20.7%)	
> 2000 $	2 (1.7%)	7 (12.1%)	
**Family history of psychiatric illness**			
Yes	42 (36.5%)	5 (8.5%)	< 0.001
No	73 (63.5%)	54 (91.5%)	
	**Mean ± SD**	**Mean ± SD**	
**Age**	48.4 ± 7.6	47.9 ± 7.4	0.67
**Duration of illness (years)**	20.6 ± 12.4	–	
**Duration of hospitalization (years)**	12.4 ± 8.5	–	
**Number of hospitalizations**	6.3 ± 5.6	–	
**Chlorpromazine equivalent dose (mg)**	1041.6 [Min: 0.5 – Max: 4502.0]		

### Testing duration

The BACS needed a mean time of 31.2 ± 5.4 min for patients and 30.1 ± 3.1 min for healthy controls. The standard battery required a mean of 42.3 ± 10.6 min for patients and 35.9 ± 3.7 min for healthy controls.

### Comparison of the mean BACS measures between patients with schizophrenia and healthy controls

Table 2 presents performances of schizophrenia patients and controls on the standard battery and BACS battery tests, including group means and standard deviations for measures of each test and z-scores for patients. When compared to healthy controls, patients with schizophrenia have significantly lower mean scores on standard battery subtests and total score (*p* < .001 for all). Also, a significant difference was found in the mean BACS measures between the two groups with a lower mean in all the BACS subtests and total score inpatients with schizophrenia compared to healthy controls (*p* < 0.001 for all).

**Table 2:** Performances of patients with schizophrenia and healthy controls on standard battery and BACS battery tests

	Patients with Schizophrenia (***N*** = 120)	Composite Z score of patients with schizophrenia	Healthy control (***N*** = 60)	***P*** value of the raw scores
	Raw score, Mean ± SD		Raw score, Mean ± SD	
**Standard battery test**	**241.8 ± 125.2**	**− 3.7**	**432.0 ± 52.0**	**< .001**
Free and Cued Recall test (RL/RI-16)	25.9 ± 14.8	− 2.2	44.3 ± 8.3	< .001
Digit span sequencing from the WAIS-IV	10.0 ± 3.4	− 0.9	13.4 ± 3.6	< .001
The Trail Making Test A (TMT-A)	150.7 ± 91.4	−4.6	48.9 ± 22.1	< .001
Verbal Fluency test	19.6 ± 8.5	−2.1	36.4 ± 8.1	< .001
Fruit category	11.1 ± 4.6	−2.3	21.5 ± 4.6	< .001
Letter B	4.7 ± 2.7	−1.2	8.2 ± 2.9	< .001
Letter F	3.8 ± 2.6	−0.9	6.7 ± 3.1	< .001
Digit Symbol Coding from the WAIS-IV	19.2 ± 14.2	− 2.0	54.3 ± 17.4	< .001
Block design test from the WAIS-IV	17.9 ± 11.5	− 1.4	32.5 ± 10.8	< .001
**BACS Battery test**	109.9 ± 47.2	−2.9	221.7 ± 38.5	< .001
List learning test	20.9 ± 9.6	− 2.1	41.1 ± 9.4	< .001
Digit sequencing	10.6 ± 5.5	− 1.9	19.3 ± 4.4	< .001
Token motor task	35.7 ± 15.0	− 2.4	70.7 ± 14.4	< .001
Verbal fluency	20.4 ± 9.2	−1.5	34.6 ± 9.2	< .001
Animal category	11.5 ± 4.7	−1.6	18.1 ± 4.1	< .001
Letter G	4.2 ± 2.7	−1.0	7.4 ± 3.2	< .001
Letter M	4.7 ± 3.4	−1.1	9.1 ± 3.9	< .001
Symbol coding	12.2 ± 12.4	−2.4	38.0 ± 10.7	< .001
Tower of London	9.9 ± 7.7	−2.1	17.9 ± 3.7	< .001

### BACS composite score profile

**Fig. 2:**
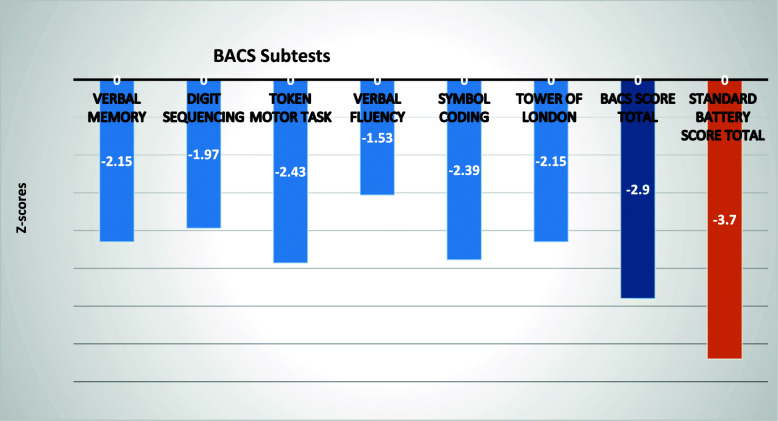
Composite scores for the BACS total score and subtests and standard battery in patients with schizophrenia standardized to healthy controls

Figure 2 shows mean composite scores for the BACS total score and subtests and standard battery in patients with schizophrenia compared to healthy controls. All differences between patients and controls were statistically significant (*p* < .001). Motor speed (*z* = − 2.43) was the most deficient function followed by attention and speed information processing (*z* = − 2.39). Also, significant differences were found between the mean composite scores from the BACS and the standard battery (− 2.9 vs. -3.7; *p* < .001).

### Correlations among BACS measures

The associations between BACS measures for patients and healthy controls are presented in Table 3. Among patients with schizophrenia, all correlations were highly significant (*p* < 0.01). Among healthy controls, all correlations were significant except correlation between token motor and tower of London.

**Table 3:** Correlations among BACS measures for schizophrenia patients and healthy controls

	VM	DS	VF	SC	TM	TL	Composite score
**In patients with schizophrenia**							
**TL**	.50***	.70***	.51***	.51***	.47***	–	.76***
**TM**	.40***	.48***	.47***	.36***	–	.47***	.74***
**SC**	.45***	.57***	.51***	–	.36***	.51***	.75***
**VF**	.65***	.67***	–	.51***	.47***	.51***	.80***
**DS**	.60***	–	.67***	.57***	.48***	.70***	.82***
**VM**	–	.60***	.65***	.45***	.40***	.50***	.76***
**In healthy controls**							
**TL**	.35**	.41**	.37**	.43**	.05	–	.45**
**TM**	.41**	.31*	.37**	.38**	–	.05	.71***
**SC**	.61***	.61***	.61***	–	.38**	.43**	.82***
**VF**	.58***	.56***	–	.61***	.37**	.37**	.79***
**DS**	.48***	–	.56***	.61***	.31*	.41**	.69***
**VM**	–	.48***	.58***	.61***	.41**	.35**	.80***

Significant correlation at: *p < 0.05, **p < 0.01, ***p < 0.001.

*VM* Verbal Memory; *DS* Digit sequencing; *VF* Verbal Fluency; *SC* Symbol Coding; *TM* Token motor task; *TL* Tower of London.

The correlation in patients with schizophrenia was controlled for duration of illness, duration of hospitalization and chlorpromazine equivalent dose

### Factor analysis of the BACS

Factor analysis for the BACS test was conducted over the schizophrenia sample. All of the items in the list could be extracted, but none of them were excluded because none of them were overly correlated (r > 0.9), had a low loading on variables (0.3), or had a low communality (0.3). The BACS subtest items converged on a single factor with an eigenvalue greater than 1, which explained 64.8% of the variance. There was a significant Bartlett’s test of sphericity (p 0.001) and the Kaiser-Meyer-Olkin estimate of sampling adequacy was 0.885 (Table 4). Furthermore, the full test had a high Cronbach’s alpha. (0.85).

**Table 4:** Factor loading of BACS measures in patients with schizophrenia

	Factor 1
Digit sequencing	.88
Verbal Fluency	.85
Tower of London	.82
Verbal Memory	.78
Symbol Coding	.77
Token motor task	.73
**Cronbach’s alpha**	.85
**Percentage of variances explained**	64.8%

### Correlations between standard battery and BACS measures

**Fig. 3:**
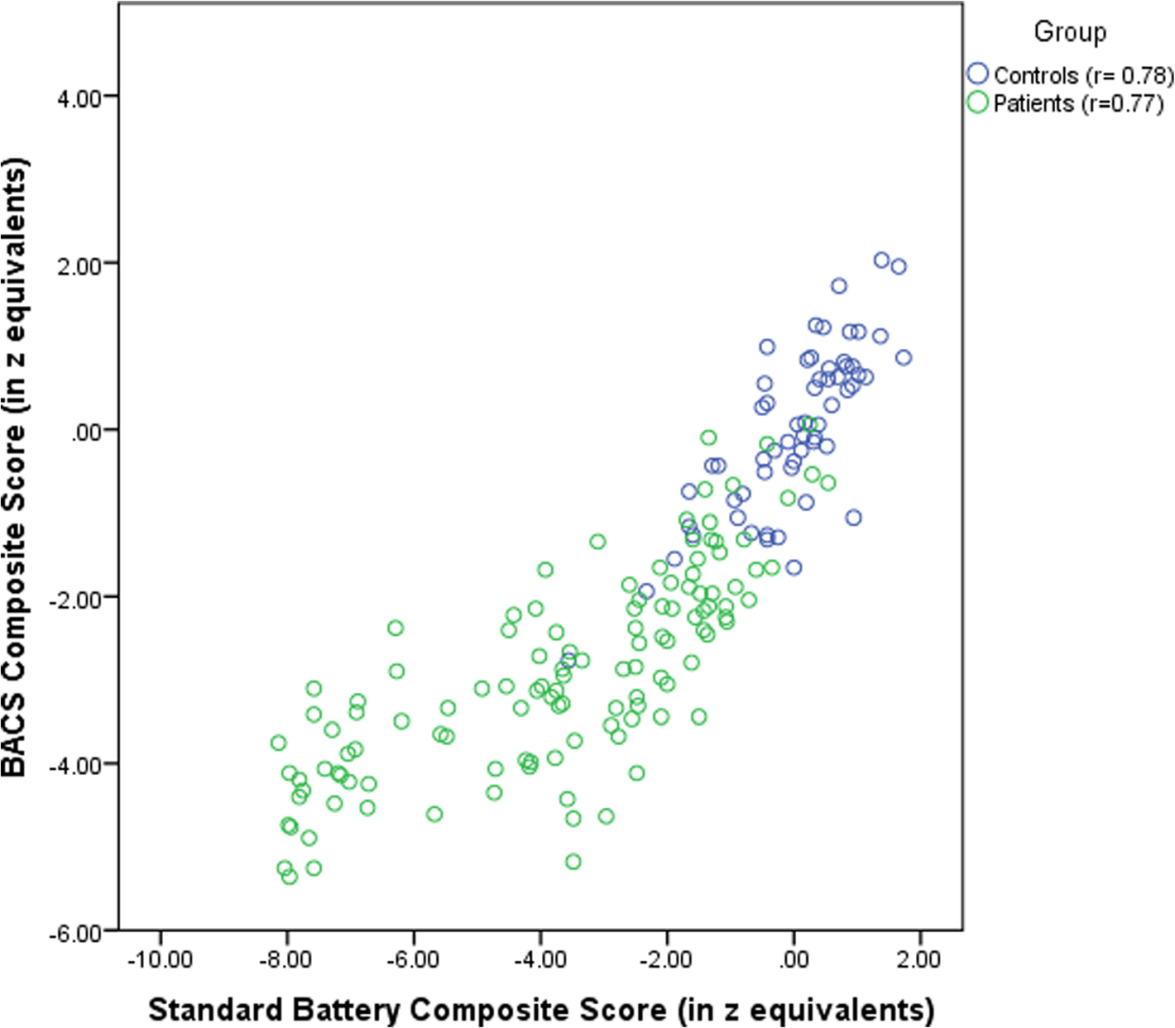
Scatterplots of BACS and standard battery composite scores for patients and controls

Table 5 shows the correlations between standard battery structures and BACS measures. In each matrix, correlations were similar between the two groups (patients with schizophrenia and healthy controls). Correlations between standard battery and BACS composite scores were 0.73 and 0.78 in patient group and control group respectively. The correlation in patients with schizophrenia was controlled for duration of illness, duration of hospitalization and chlorpromazine equivalent dose. Figure 3 demonstrate the individual data points from this correlation.

**Table 5:** Pearson correlations between standard battery domains and BACS measures

Standard Battery	BACS measures						
	VM	DS	TM	VF	SC	TL	Composite BACS score
**In patients with schizophrenia**							
Free and Cued Recall test (RL/RI-16)	.53***	.57***	.16	.57***	.40***	.43***	.54***
Digit span sequencing from the WAIS-IV	.46***	.71***	.36***	.59***	.63***	.55***	.69***
The Trail Making Test A (TMT-A)	.36***	.60***	.40***	.52***	.55***	.41***	.61***
The Controlled Oral Word Association Test; Category Instances	.59***	.65***	.35***	.80***	.50***	.45***	.70***
Digit Symbol Coding from the WAIS-IV	.47***	.55***	.42***	.50***	.81***	.42***	.70***
Block design test from the WAIS-IV	.47***	.58***	.31**	.50***	.45***	.58***	.60***
**Composite standard battery score**	.49***	.70***	.43***	.64***	.65***	.51***	.73***
**In healthy control**							
Free and Cued Recall test (RL/RI-16)	.64***	.35**	.22	.53***	.54***	.26	.58***
Digit span sequencing from the WAIS-IV	.48**	.54***	.35**	.42**	.61***	.24	.60***
The Trail Making Test A (TMT-A)	.50***	.50***	.21	.50***	.58***	.58***	.60***
The Controlled Oral Word Association Test; Category Instances	.53***	.55***	.34**	.73***	.52***	.26*	.66***
Digit Symbol Coding from the WAIS-IV	.57***	.51***	.28*	.43**	.83***	.28*	.67***
Block design test from the WAIS-IV	.27	.37**	.11	.23	.38**	.34**	.34**
**Composite standard battery score**	.68***	.64***	.32*	.63***	.81***	.51***	.78***

Significant correlation at: *p < 0.05, **p < 0.01, ***p < 0.001.

*VM* Verbal Memory; *DS* Digit Sequencing; *TM* Token Motor Task; *VF* Verbal Fluency; *SC* Symbol Coding; *TL* Tower of London.

The correlation in patients with schizophrenia was controlled for duration of illness, duration of hospitalization and chlorpromazine equivalent dose

### ROC analysis

**Fig. 4:**
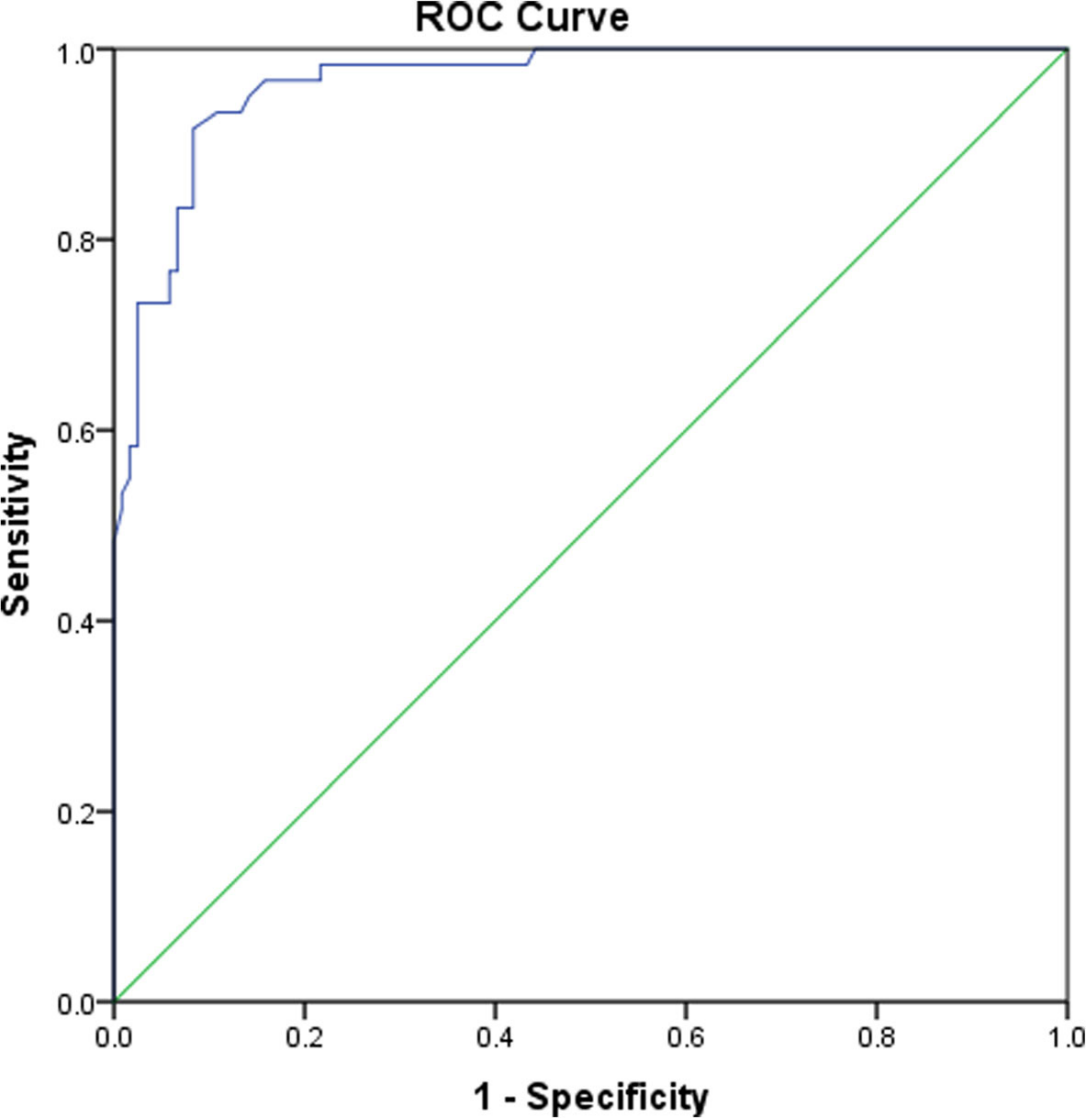
ROC curve of the BACS for the predicted probability for being either identified as a patient or a healthy control. Area under the curve = .96 [.94–0.99] (*P* < .001); at value = 163 (z score value = − 1.51), Se = 93.3% and Sp = 86.7%

A ROC analysis was used to investigate the function of the BACS composite score in distinguishing patients with schizophrenia from healthy controls. The area under the ROC was .96 (confidence interval = .94–.99, *P* ≤ .001), sensitivity was .93 and specificity was .86 with the cut-off value of 163 (z score = − 1.51). This finding suggests that the BACS composite score has a high capacity to distinguish between schizophrenia patients and healthy controls (Fig. 4).

## Discussion

The Arabic version of the BACS demonstrated strong concurrent validity with a standard battery of cognitive tests, high internal consistency with a one-factor structure, and the capacity to distinguish patients with schizophrenia disorders from healthy controls. In patients, it took 31.2 min to administer BACS almost equal to the original BACS where the completed time was 34.2 min [14]. Testing period could be shortened by the administrators as they become more familiar with the administration of tests. Administration durations of the BACS in patients and controls were similar displaying a consistent performance of the administrator. Duration difference between administration of BACS (31.18 min) and of standard battery (42.33 min) in patients was 11.15 min, which could be explained by the longer duration required by some standard battery tests (for example RL/RI-16 test).

Good concurrent validity was found between BACS and standard battery composite scores (*r* = 0.73) similar to correlations found in the original BACS article [14]. The BACS neurocognitive battery is a useful tool for measuring global cognitive performance of schizophrenia patients. Concerning subtests of the BACS among patients with schizophrenia, we also found a good correlation (r > 0.71) for digit sequencing, symbol coding and verbal fluency but less good for tower of London (*r* = 0.58). Lower correlations were found for verbal memory (*r* = 0.53) and motor speed tests (*r* = 0.40). Similar results were found for the motor speed tests in the original version [14], French [15], German [16], Persian [21] and Spanish [17] versions. The low correlations between the Token Motor Task and the TMT A may be explained by the fact that both tests evaluate slightly different facets of cognitive functions [21]. The TMT-A is used as an indicator of visual scanning, graphomotor speed and executive function [34], however the token motor task is used to measure a rapid motor coordination task. For the verbal memory task, a methodological difference could exist in assessment of episodic memory between list learning test and RL/RI-16 tests [35]. Also, these two tests had not yet been adapted and validated into the Lebanese language, as alternate words might facilitate the assessment as difficulty in remembering the required words might exist.

A one-factor solution underlying the BACS subtests was discovered using factor analysis on the Arabic-BACS, which explained 64.8% of the instrument overall variance. Similarly, a unique factor structure was found in the Spanish [17] and Persian versions [21]. Although the original BACS [14] and the Japanese BACS [20] discovered a three-factor solution, and the French BACS [15] showed a two-factor solution, they all discovered that a single factor explained the majority of the variance. Different sample sizes and variations in clinical symptoms among participants may explain the disparities between the studies. We have found a strong level of internal consistency, as shown by a high Cronbach’s alpha and statistically meaningful correlations between the scale’s individual items. The BACS can be used to measure general cognitive function in schizophrenia, according to our results.

ROC analysis revealed that the BACS composite score at a level of − 1.51 had high level of sensitivity and specificity to differentiate patients with schizophrenia and controls. In line with the German [16], Chinese [19] and Persian [21] versions, our findings suggest that the Arabic-BACS can be used to distinguish patients from controls based on neurocognitive function.

All the subscales and total scale scores of the Arabic-BACS differed significantly between patients and controls. We also noted that mean BACS global score and subscales scores had high deficiency as compared to the original version [14]. Also, the cognitive task that was mostly deficient in our patients was the motor speed followed by attention and processing speed. However in the original article the most deficient cognitive task was verbal memory followed by attention and processing speed [14]. It is well recognized that psychomotor task is among most affected cognitive domain in schizophrenia [36]. Our findings could be clarified by the fact that selected patients were institutionalized for a long period and might have more cognitive impairment than patients selected from outpatient clinics.

## Limitation

Several limitations have been found in this study. First, because of the limited sample size, the study’s findings could not be generalized to the whole population and patients were selected from one single site. Second, information bias might have occurred since accurate details could not be provided from participants in a face-to-face interview. Third, test retest was not assessed. Also, the order of the tests administration was not randomized which could have generated a confounding bias. Finally, further comparisons need to be made for the Verbal Memory task. Further studies including evaluations of various schizophrenic subgroups would be needed to fully validate the BACS (first episode populations, treatment refractory schizophrenic patients and geriatric patients).

## Conclusion

The psychometric properties of the Arabic version of the BACS were adequate, with high internal consistency, appropriate concurrent validity, and good overall discriminant validity. In daily psychiatry clinical practice and in research studies, we suggest that Arabic BACS is a reliable and useful tool for measuring cognitive performance in inpatients with schizophrenia. However, it is unclear if the results of this study can be applied to other Arabian communities that speak different dialects. In order for the BACS to fulfill all of the criteria of a good cognitive tool, normative data from a healthy population should be collected in the future.

## Data Availability

Data can be made available under reasonable request form the corresponding author.

## Abbreviations

BACS: Brief assessment of cognition in schizophrenia
SD: Standard deviation
MATRICS: Measurement and treatment research to improve cognition in schizophrenia
RBANS: Repeatable battery for the assessment of neuropsychological status
CANTAB: Cambridge neuropsychological test automated battery
WAIS: Wechsler adult intelligence scale
HPC: Psychiatric hospital of the cross
DSM-5: Diagnostic and statistical manual of mental disorders, fifth edition
USD: United States dollar
TMT-A: Trail making test A
SPSS: Statistical package for social sciences
VM: Verbal memory
DS: Digit sequencing
VF: Verbal Fluency
SC: Symbol coding
TM: Token motor task
TL: Tower of London
ROC: Receiver operating characteristic curve
